# Reviewed article: Almeida ALS, Sousa TMS, Caldeira TCM, Claro RM. Association between adherence to the Food Guide golden rule and health characteristics among adult Brazilian women: a cross-sectional study with VIGITEL data, 2018-2021. Epidemiol Serv Saude. 2025:34;20240232.

**DOI:** 10.1590/S2237-96222025v34e20240232.a

**Published:** 2025-04-11

**Authors:** Cláudia Tramontt

**Affiliations:** 1 USP, Nutrição

**Table:** 

Rating	Excellent	Good	Average	Below average	Poor
Interest		✓			
Quality			✓		
Originality			✓		
Overall		✓			

**Table:** 

Questionnaire	Yes	No	Not applicable
Does the manuscript contain new and significant information to justify publication?	✓		
Does the Abstract (Summary) clearly and accurately describe the content of the article?	✓		
Is the problem significant and concisely stated?	✓		
Are the methods described comprehensively?	✓		
Are the interpretations and conclusions justified by the results?	✓		
Is adequate reference made to other work in the field?	✓		

## Manuscript Structure:

Length of article is: Adequate

Number of tables is: Adequate

Number of figures is: Adequate

Please state any conflict(s) of interest that you have in relation to the review of this paper (state “none” if this is not applicable): None

## Comments

Sugiro que os autores destaquem a originalidade do trabalho, relacionando-o com a literatura mais atual existente, explicando sua contribuição para o campo de estudo e o que este trabalho adiciona de novo. Expliquem qual a relação com a prática, uso/aplicabilidade, correspondência com o contexto dos usuários atendidos e os profissionais de saúde dos serviços do SUS.

Comentários específicos:

Introdução:

2º parágrafo: o que os autores querem dizer na definição de alimentos in natura e minimamente processado com “principalmente para preservação e melhoria da disponibilidade, segurança e sabor.” ? da mesma forma, sugiro rever a definição de alimentos ultraprocessados utilizando a definição mais atualizada presente na seguinte referência:

Monteiro CA, Cannon G, Levy RB, Moubarac JC, Louzada ML, Rauber F, Khandpur N, Cediel G, Neri D, Martinez-Steele E, Baraldi LG, Jaime PC. Ultra-processed foods: what they are and how to identify them. Public Health Nutr. 2019 Apr;22(5):936-941. doi: 10.1017/S1368980018003762. Epub 2019 Feb 12. PMID: 30744710; PMCID: PMC10260459.

4º parágrafo: “A população feminina é reconhecida por apresentar práticas alimentares mais saudáveis além de uma menor prevalência de fatores de risco à saúde, como tabagismo, consumo regular de bebidas açucaradas e consumo abusivo de álcool. ” - Não está claro. A referência 8 não parece trazer essa evidência.

“No entanto, as disparidades socioeconômicas de gênero resultam em menores níveis de educação e renda, levando a população feminina a um menor acesso a alimentos de alta qualidade e maior risco de insegurança alimentar. 9 Além disso, lares chefiados por mulheres representam a maioria no Brasil (50,8%),10 com as mulheres equilibrando múltiplas responsabilidades, incluindo ser a principal provedora de alimentos e refeições em seus domicílios, influenciando significativamente o estado nutricional dos membros da família, especialmente crianças.”

- o que seriam alimentos de “alta qualidade”? eles são mais caros? se mulheres possuem menor renda como elas são a principal provedora de alimentos e refeições em seus domicílios? Está confuso. Sugiro fundamentar melhor a argumentação.

Métodos:

- “O consumo de cada subgrupo de alimentos in natura e minimamente processados adicionou 1 ponto à pontuação” - sugiro usar “escore” ao invés de pontuação.

-Não tive acesso a figura suplementar.

“A prevalência de diabetes, hipertensão e depressão foi determinada com base em uma resposta afirmativa à pergunta:” - incluir que em qualquer ano da pesquisa.

Resultados:

“RO” - explicar a primeira vez que utilizar. Discussão:

O primeiro parágrafo está repetitivo e um pouco redundante. Sugiro, se quiserem enfatizar os resultados, que os apresente de forma diferente, como por exemplo, abordando quantas vezes o risco foi maior nos grupos comparados.

- linha 49 da página 12: em relação ao feijão, cabe trazer a justificativa/informação de que ele é um importante marcador de alimentação saudável, preditor da qualidade da dieta.

- linha 6 pagina 13: Essa afirmação precisa ser melhor explicada e referenciada, trazendo estudos que evidenciam isso em lares brasileiros - se houver.: “Os padrões alimentares da população feminina desempenham um papel importante nos desfechos de saúde de seus lares, dado que as mulheres também desempenham um papel fundamental no planejamento das refeições familiares”.

- pág 14 linha 20: no trecho “para fortalecer a produção de alimentos locais, e medidas que regulam a publicidade de alimentos, campanhas de educação nutricional e iniciativas que promovem a alimentação saudável.” De quais medidas, campanhas e iniciativas estão falando? Sugiro trazer o debate mais atual e a aplicabilidade no campo das políticas públicas brasileiras.

- linha 29: em relação às informações autorrelatadas por entrevista telefônica - apontar as limitações desse método. Explique como os vieses foram controlados e como a validade interna do estudo foi assegurada.

- linha 52, no trecho “a adesão às práticas alimentares recomendadas pelo Guia Alimentar para a População Brasileira tem sido objeto de estudo e diversas pesquisas”. Apesar do “diversas pesquisas” os autores trazem apenas uma referência, que fala sobre validação. Se os pontos de corte não foram validados, discuta as limitações dessa falha.

- por fim, sugiro que os autores reflitam e consolidem melhor as justificativas e achados em relação ao que este estudo traz de novo? Explique as implicações práticas das descobertas, discutindo sua relevância para a prática clínica, políticas de saúde ou futuras pesquisas.

